# Assessing Evolutionary Significant Units (ESU) of the Endangered Freshwater Pearl Mussel (*Margaritifera margaritifera*) in Southeast Norway on the Basis of Genetic Analysis

**DOI:** 10.3390/genes11091061

**Published:** 2020-09-08

**Authors:** Arne N. Linløkken, Silje Garlie, Wenche Johansen, Robert C. Wilson

**Affiliations:** Faculty of Applied Ecology, Agricultural Sciences and Biotechnology, Inland Norway University of Applied Sciences, N-2418 Elverum, Norway; SiljeGarlie@yahoo.no (S.G.); Wenche.Johansen@inn.no (W.J.); Robert.Wilson@inn.no (R.C.W.)

**Keywords:** freshwater bivalves, genetic diversity, immigration, landlocked, isolation, threats

## Abstract

A total of 312 specimens of freshwater pearl mussel (*Margaritifera margaritifera*) were sampled from 11 populations, located in four different river systems in Southeast Norway, and analyzed for 11 simple sequence repeat (SSR) (microsatellite) markers. All study populations have landlocked brown trout (*Salmo trutta*) as the only possible host. Several populations had experienced recruitment failure, probably due to low pH (about 6.0) and calcium concentration. STRUCTURE clustering analysis revealed two genetic clusters, of which one cluster occurred mainly in the western river systems, and totally dominated in one population (Fallselva (A-FAL)) that had higher genetic diversity than the others. Cluster 2 completely dominated in the populations of the eastern river systems, and all of them had low genetic diversity. Bottleneck events were indicated in all populations and the inbreeding coefficient *F*_IS_ was significant in all populations, except for the southernmost population (Sørkedalselva (B-SØR)), which was the only population with genotypes in Hardy–Weinberg equilibrium. *F*_IS_ were especially high in the populations of the eastern river systems, and maximum shell length was negatively correlated to *F*_IS_. If artificially breeding and stocking should become necessary for future preservation, it should be based on single populations; alternatively, the eastern populations should be based on cross-breeding of populations within the cluster to increase their genetic diversity.

## 1. Introduction

Freshwater bivalvia is a group with several endangered species, and the freshwater pearl mussel (*Margaritifera margaritifera*) is one of the most threatened [1,2,3,4]. It is consequently on the European Red List [5]. The freshwater pearl mussel is distributed from the arctic and temperate regions of western Russia, through Europe to the northeast North America [6], and their preferred habitat is oligotrophic streams where they play an ecological role by filtering water [7,8,9,10]. In populated areas, mussel habitats are often subject to physical interventions, and in some areas they are affected by eutrophication and sedimentation [11], in other areas by acidification [12]. Drastic declines and extinctions have taken place in numerous locations [3,12,13,14], and in addition to habitat deterioration, exploitation by humans for harvesting pearls has put the species at special risk [15].

In the management of biological resources, conservation of threatened species is an important issue, and extinction due to habitat loss may be a scenario with survival in captivity as the worst case alternative [16]. Several concepts are used in management and research, such as conservation units (CU) [17], which are identified in two levels [18], as management units (MU) [19] representing demographically independent units, and evolutionary significant units (ESU) [19,20,21] representing evolutionary/ecological components of the species as a whole. The concepts are used on intraspecific levels, commonly on populations regarded as especially important. When aiming to maintain the evolutionary processes and viability of populations, the ESU concept is the most relevant. Several frameworks for defining ESUs have been proposed, and most of them can be characterized in terms of diversity, isolation, and adaptation [21]. Isolated, viable populations and their habitats should be protected, but in addition, long-term viability demands genetic diversity for adaptive evolution, and genetic analysis to describe the populations’ present state is necessary to assess relevant ESU and MU [21,22,23].

Adult freshwater pearl mussels are more or less stuck in their habitat, with a parasitic larval stage, and depend on their host fish, salmon (*Salmo salar*) or brown trout (*Salmo trutta*), to reproduce and to settle in new areas [15]. Habitat destruction and catastrophic events may eradicate local host fish populations and prevent recruitment of the mussel, and particularly in landlocked parts of river systems, the mussel will be prone to extinction. However, longevity of the species, spanning 100 years or even more, allow populations to survive for decades without host fish being present [24,25].

The freshwater pearl mussels’ low mobility and the females’ ability to turn to hermaphroditism at low densities [26] make small persisting populations vulnerable to inbreeding, and low genetic diversity due to inbreeding has been reported, especially for populations on the edge of the species distribution area [27], as compared with those that are more centrally located [28]. Populations in landlocked systems therefore show lower genetic diversity but exhibit heightened genetic structure when compared with populations hosted by anadromous salmonids [29]. As inbreeding is a potential threat to populations’ sustainability, conservation strategies may benefit from inputs from population genetic studies [22,28,30]. Inbreeding is shown to negatively affect shell length and recruitment for scallops in captivity, but these effects can be counteracted by selective breeding [31,32,33,34]. 

In Norway, approximately 560 locations harbor, or have harbored, freshwater pearl mussels, but approximately 25% of these are lost [14], and the species is most abundant in rivers with salmon and sea trout [14,29,35]. In central South Norway, the species occurs in high densities in some landlocked systems northwest of the Oslo Fjord, in the counties of Viken and Telemark, whereas it is less abundant in landlocked river systems in the central and easternmost part of South Norway. A major part of this eastern area is drained by the Glomma river system, including the Lake Mjøsa, and freshwater pearl mussel is lacking in the central and upper part of the catchment [14].

Historical records confirm that mussels from the easternmost part of South Norway were collected and sold until about 100 years ago, but in small amounts compared to what was sold from the neighboring County of Viken (including the former County of Buskerud) farther west and south [36,37]. This probably reflected a lower abundance of mussels in the easternmost part of South Norway as compared with that in Viken County, also in past decades. Early in the 20th century, groups of tramps were observed collecting mussels in heaps on the riverbanks in the County of Buskerud before they killed and examined them, and this took place in rivers where the mussels still are abundant. Governmental authorities advocated the introduction of the mussel to areas where they were lacking, but no written sources are found to confirm whether this was performed (Simonnæs 1927, cited in [36]).

As the freshwater pearl mussel populations in the southeastern part of South Norway are sparse due to weak reproduction and isolation, several populations are regarded as threatened [14]. Mussels in oligotrophic streams in this area are prone to acidification [38], with pH below the species optimal at pH = 6.3–8.0 [39]. Although adult specimens may survive episodes of pH < 5.0, the glochidia larvae are more vulnerable [40]. To secure their preservation, supportive measures must be considered, and this study aimed to explore the genetic structure and diversity by means of simple sequence repeats (SSRs) of mussels from 11 sampling locations situated in four main river systems. Most of the locations are at the northern edge of the species’ distribution in the inland district of South Norway, and the populations differ in abundance and viability. In addition, due to an established theory of immigration history of fish in southeast Norway [41], groups of populations may have different ancestry. Conservation measures such as semiartificial infections of host fish and supportive breeding must be based on local strains [28], and this study explored genetic structure to reveal the populations’ ancestry, degree of isolation, and their genetic diversity to assess inbreeding and the populations’ evolutionary potential. This was further meant to identify the local evolutionary significant unit (ESU) [19,28] of the species. Potential inbreeding effects on recruitment and shell length of populations were also considered.

## 2. Materials and Methods

### 2.1. Study Area

**Figure 1:**
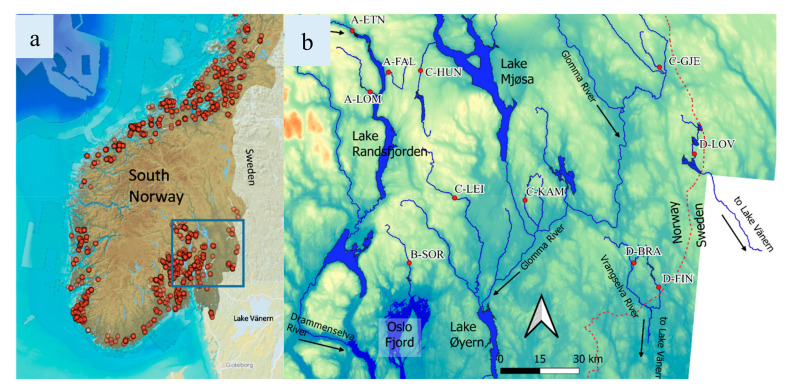
(**a**) Distribution of freshwater pearl mussel (*Margaritifera margaritifera*) in South Norway, according to the Norwegian Biodiversity Information Centre (NBIC) 2020 [43] (the blue frame in figure (**a**) sections figure (**b**)) and (**b**) a map of the study area with the 11 sampling locations (right).

The sampling locations were chosen in known freshwater pearl mussel locations in four main river systems (the western A and B, and the eastern C and D) in Southeast Norway (Figure 1), representing the border of the species distribution in the inland district. In total, 312 specimens of freshwater pearl mussels were sampled from 11 different locations. The upper marine limit in the area is approximately 200 m above sea level (m a.s.l.), and the sampling sites are situated at 150 to 304 m a.s.l. pH ranged from 5.3 to 7.6 (the low values in spring) and [Ca] from 1.4 to 16.0 mg/L. With the exception of one location (River Hunnselva, C-HUN), all the streams exhibited low conductivity and low to moderate alkalinity (Table 1). Due to acidification, lakes upstream the following sampling locations: Lomsdalselva (A-LOM), Fallselva (A-FAL), Leira (C-LEI), Kampåa (C-KAM), Bråtåa (D-BRÅ), and Løvhaugsåa (D-LØV), were lime treated, i.e., limestone powder was added to increase pH, from the early1990s, but all treatments were terminated before 2013 [42].

Larsen and Magerøy [14] reviewed freshwater pearl mussel surveys from Norway, covering more than 400 populations including the populations in this study, and categorized the populations’ statuses. This was based on six criteria of importance for the long-term survival of populations: (1) population size, (2) average density, (3) prevalence, (4) smallest shell, (5) proportion of shell lengths <20 mm (assumed to represent age groups <10 years), and (6) proportion of shells <50 mm (assumed to represent age groups <20 years). More details are given in Table S1. Populations are given 0–6 points of each criteria, and the points of each criterion are added for the population. Living populations may attain a total of 4 to 36 points, i.e., the former means that old specimens are present (0 means extinct), and the latter means six points of each criterion. The populations of the present study scored from 6 to 22 points, with a mean of 11.3 (Table 1), and four scored ≤ 7 and were characterized as threatened [14]. The study samples were not representative of the smallest specimens as they were avoided for DNA sampling.

The streams Lomsdalselva (A-LOM), Etna (A-ETN), and Fallselva (A-FAL) drain to the Lake Randsfjorden and further to the Drammenselva River (A) and the Oslo Fjord. There is one migration obstacle to fish downstream (130 m a.s.l.) of the Lake Randsfjorden and one obstacle (250–275 m a.s.l.) below the sampling site of A-FAL. All the A populations (the westernmost populations hereafter) had recruitment failure, and measures were conducted to improve recruitment by breeding larvae in artificial streams for stocking after four years [14]. The population in the Sørkedalselva River (B-SØR, the southernmost population), with an outlet to the northern part of the Oslofjorden, has fairly good recruitment [44], and there is a migration obstacle (140 m a.s.l.) downstream the sampling site.

In the large Glomma river system (C), relatively few locations harbor freshwater pearl mussels, and most of them are sparse with poor recruitment [44]. Samples were collected from four streams in the river system, mentioned from the west: Hunnselva (C-HUN), Leira (C-LEI), Kampåa (C-KAM), and Gjerda (C-GJE). There is a migration obstacle to fish downstream of the sampling site in C-LEI at 225 m a.s.l., and one obstacle in C-HUN at 150 m a.s.l., close to Lake Mjøsa (120 m a.s.l.). Natural recruitment was lacking in C-HUN, whereas there was weak recruitment in C-LEI and C-GJE, and fairly good recruitment in C-KAM, with >25% of the specimens <60 mm (<20 years), coinciding with a period of liming [45]. There are several migration obstacles to fish in the Glomma River downstream of the stream inlets of Leira and Kampåa, both natural waterfalls and power plant dams, and salmon are only present in the lowermost 17 km of the Glomma River, downstream of the waterfall Sarpsfossen.

The streams Finnsrudåa (D-FIN), Bråtåa (D-BRÅ), and Løvhaugsåa (D-LØV) (hereafter the easternmost populations) draining into the Lake Vänern, and the recruitment was characterized as good in D-FIN, whereas recruitment was weaker in D-BRÅ and D-LØV, and was probably positively affected by liming of upstream lakes in the 1990s [14,46,47].

### 2.2. Sampling and Analysis

From 2009 to 2011, haemolymph samples (0.1–0.3 mL) were collected from the foot with 1 mL syringes attached to 1.00 × 40 mm 21Gx2” sterican needles and stored in Eppendorf tubes with 400 μL RNALater (Ambion, Austin, TX, USA). We attained permission to sample mussels from the local County Environmental Administration (Fylkesmannen in Hedmark, reference 2016/311). Bivalvia is not included in the Norwegian Act of animal welfare, but the collected mussels were treated with care and kept in fresh water, except for 1 minute or so during sample collection, and put back at the sampling site. The use of a needle nevertheless represents a risk of injury, and during 2011–2016, samples were taken with the non-invasive mucus sampling with Q-tips (overlapping the first method in C-GJE) gently rolled on the foot of the mussel, and stored dry and cool in Eppendorf tubes until freezing (−20 °C) as soon as possible. Nuclear DNA was isolated with chelex [48], and 11 SSR markers were PCR-amplified in two separate multiplexes: (multiplex-1, Table S2) Mm2209, Mm2230, Mm2233, Mm2235, Mm2236, Mm2238 [49] and (multiplex-2) MarMa3116, MarMa3621, MarMa4277, MarMa4315, and MarMa5167 [50]. Data were analyzed and evaluated with the GeneMapper (ver. 4.0, Thermo Fisher Scientific, Waltham, MA, USA) software. Randomly chosen DNA samples (≈10%) were subjected to a second round of PCR and electrophoresis to assess the consistency of the assay. The mean scoring success across samples was 96.9% (± SD = 1.70), ranging from 94.5 to 99.7%. The primary results are given in Table S3.

### 2.3. Statistical Analysis

The software STRUCTURE 2.3 [51] was used to infer the most likely number of population clusters (*K*) constituting each sample, and the number of clusters was assumed to correspond to the number of ancestor populations [51]. Each individual was assigned a membership coefficient (*Q*) for each inferred cluster. Ten different runs were performed for each *K* (1–12, i.e., 1–n + 2) simulated, assuming an admixture model. A burn-in period of 50,000 iterations and a Monte Carlo Markov Chain (MCMC) of 50,000 iterations were used. The optimum number of clusters *K* was determined by means of the STRUCTURE-HARVESTER software [52], as described by Evanno et al. [53]. The estimated cluster membership coefficient matrices for the best fitted *K* was permuted so that all replicates have as close a match as possible using the CLUMPP software version 1.1.2. [54].

Genetic diversity indices including allele frequency, expected (*H*_E_) and observed heterozygosity (*H*_O_), number of alleles per locus (*A*_L_), inbreeding coefficient (*F*_IS_), and deviations from Hardy–Weinberg equilibrium (HWE) were explored by means of the web based GenePop software. Linkage disequilibrium and indication of loci under selection (with Markov chain parameters set at the maximum dememorization number and maximum number of iterations per batch (10,000) for 1000 batches) were explored by means of the ARLEQUIN 3.1 software [55]. Allele richness (*A*_R_) was calculated (for sample size 20) by means of the HP-Rare 1.1 software [56], and relatedness was explored by means of the ML-Relate software [57]. The GenAIEx 6.5 Excel ad in [58] was used to calculate pairwise genetic differentiation *F*_ST_ [59] and Nei’s differentiation index (*Nei*D) [60]. A dendrogram-based hierarchical clustering of allele frequencies was constructed by means of the R-software package ape [61].

The BOTTLENECK 1.2.02 software [62] was executed using an infinite allele mutation model (IAM; the least appropriate for SSR data [63]), a stepwise mutation model (SMM), and a two-phase mutation model (TPM, assumed to be the most realistic model for SSR [64]). Populations exhibiting a significant number of loci with heterozygote excess by means of a Wilcoxon sign-rank test have likely undergone a recent population bottleneck event. A second method to reveal bottleneck events is calculation of the Garza–Williamson modified index [65] across loci by means of the ARLEQUIN 3.5.1 software. This index is a ratio *M* = the number of alleles (k) divided by the range in allele size (r), on the basis of the assumption that the number of alleles declines faster than the range in allele size during a bottleneck. Any dataset with seven loci or more with *M* < 0.68 can be assumed to have gone through a recent reduction in size, i.e., a bottleneck event [65].

## 3. Results

In total, 312 specimens of freshwater pearl mussels from 11 different streams were analyzed, and the number of alleles recorded for 11 SSR markers ranged from 15 to 39. No significant linkage disequilibrium nor indication of selection were observed at any locus after Bonferroni correction.

### 3.1. Genetic Structure

**Figure 2:**
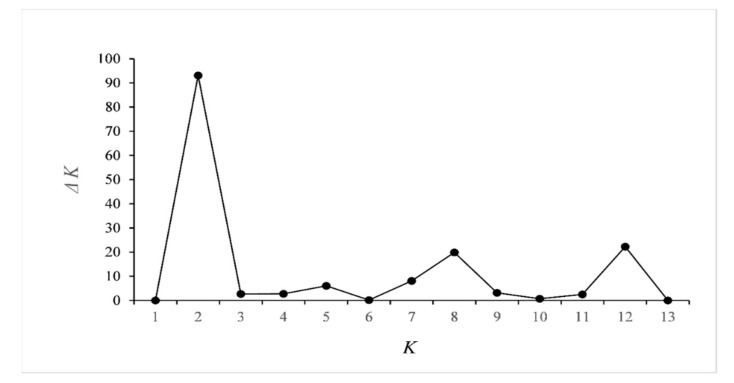
*ΔK* plotted on number of clusters *K* by means of the STRUCTURE-HARVESTER software.

**Figure 3:**
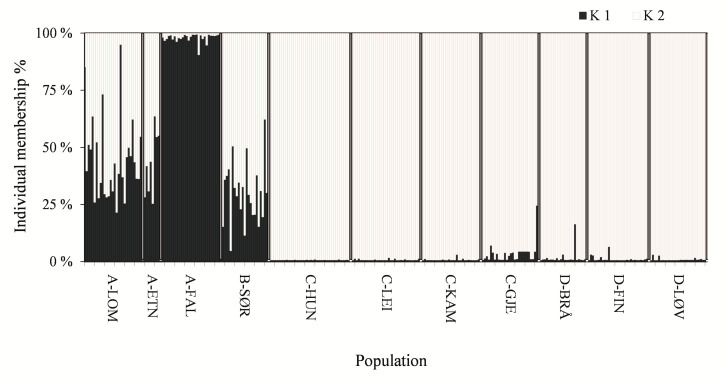
Summary plot of the estimated individual membership coefficients of each cluster (K1 and K2). Each individual is represented by a single vertical line broken into segments, with lengths proportional to each of the *K*-inferred clusters.

**Figure 4:**
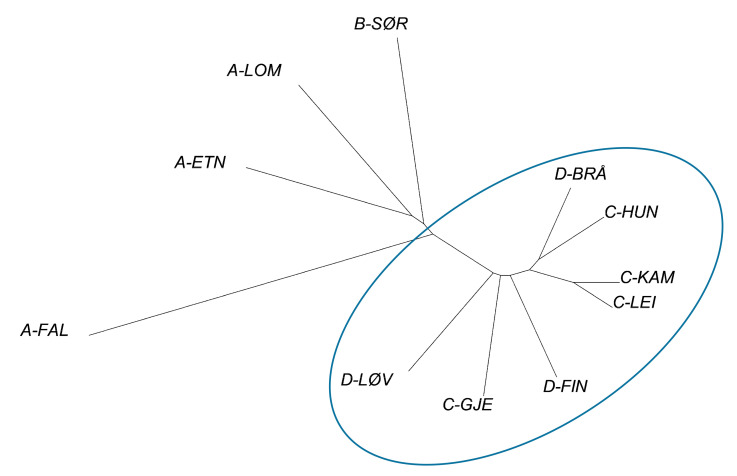
Node-less phylogenetic tree based on allele frequencies of the 11 study populations. The eastern populations are encircled.

Pairwise differentiation, expressed as *F*_ST_ and *Nei*D, was significant in all pairs, except for the C-LEI/C-KAM pair (Table 2). The STRUCTURE-HARVESTER analysis suggested two main clusters (Figure 2), of which cluster 1 completely dominated in the isolated A-FAL population, and comprised 43–45% of the individuals of the other westernmost populations and 30% of the southernmost population (B-SØR) (Figure 3). Cluster 2 comprised 96–99% of the individuals of the eastern populations. From the node-less phylogenetic tree based on allele frequencies, the A-FAL population stands out as an exclusive group, with the rest of the western populations being relatively closely interrelated (Figure 4). The eastern populations, appearing as the lower right ranch of the phylogenetic tree, appear as admixed between the river systems. The two samples C-LEI and C-KAM are closely related, as demonstrated by the non-significant *F*_ST_ and *Nei*D index. C-HUN and D-BRÅ are also relatively close, although the sampling sites were the geographically most distant among the eastern populations. Likewise, the C-GJE, D-FIN, and D-LØV were relatively close. The genetic differentiations showed no clear relationship to geographic distance, except for the “neighbors” A-LOM and A-ETN. The A-FAL and the C-HUN, sampled from two streams with neighboring catchments but of different main river systems, were found to be substantially differentiated.

### 3.2. Genetic Diversity

**Figure 5:**
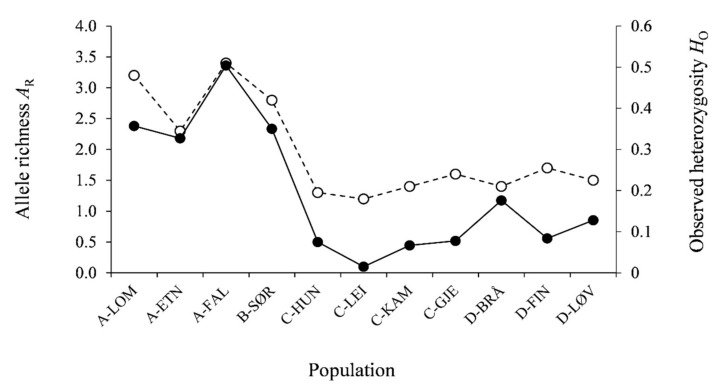
Allele richness (*A*_R_, dotted line) and observed heterozygosity (*H*_O_, solid line) of the 11 study populations.

The genetic diversity was generally higher in the western compared to the eastern populations (Table 3, Figure 5). Of the western populations, 10 to 11 loci were polymorphic, while only four to seven loci were polymorphic in the eastern populations. The number of alleles per locus (*A*_L_) and allele richness (*A*_R_) ranged from 1.4 to 4.1 and 1.2 to 3.3, respectively, and *A*_L_ was ≥2.4 in the western and ≤1.8 in the eastern populations, and correspondingly *A*_R_ was ≥2.7 in the western and ≤1.7 in the eastern populations. Expected heterozygosity (*H*_E_) was ≥0.586 in the western and ≤0.422 in the eastern populations, and the homozygote excess was significant in all samples, except for in B-SØR, which was in Hardy–Weinberg equilibrium (HWE). A-LOM and A-FAL had the highest numbers of private alleles, 11 and 9, respectively, whereas the B-SØR sample had five private alleles. In the samples from the eastern river systems, there were null to four private alleles across samples.

**Figure 6:**
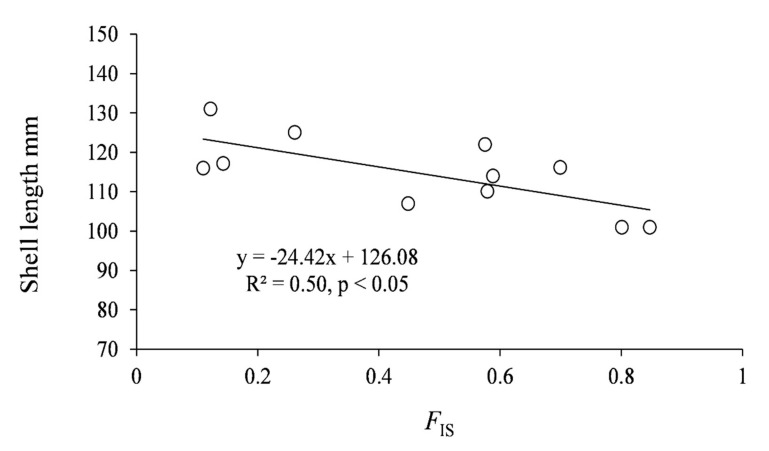
Maximum shell length within sample plotted on the inbreeding coefficient *F*_IS_.

The inbreeding coefficient *F*_IS_ estimates were significant in all samples, except for the B-SØR population, and ranged from 0.101 to 0.170 in the samples from the western river systems. *F*_IS_ was considerably higher in the samples from the eastern river systems, ranging from 0.465 to 0.825, and the maximum shell length within samples was negatively correlated to *F*_IS_ (Figure 6, *F*_1,9_ = 8.73, *p* < 0.05). There was no correlation between *F*_IS_ and status category (Table 1 and Table 3) (*p* > 0.05).

The proportion of probable siblings was also considerably lower among the western than among the eastern populations (Table 4).

Due to heterozygote deficiency in all populations, the method employed to detect recent bottleneck events as heterozygote excess using the BOTTLENECK software was not suited. Nevertheless, the mean Garza–Williamson modified index varied from 0.017 to 0.23 and was < 0.50 at all loci, except at two markers (Mm 2233 and MarMa 3621) in the A-LOM, A-FAL, and B-SØR samples (Table S3), i.e., recent bottleneck events were indicated in all populations.

## 4. Discussion

### 4.1. Genetic Structure and Probable Origin of the Study Populations

STRUCTURE analysis of 312 individuals of freshwater pearl mussels from four main river systems suggested two clusters, of which cluster 1 totally dominated in one of the westernmost populations, the isolated and elevated A-FAL population (in mean 98% of the individual genomes). Cluster 1 also made up 30 to 45% of individuals of the other western populations, whereas the eastern populations were completely dominated by cluster 2, comprising 96 to 99% of the individual genomes. This corresponded to the node-less phylogenetic tree, with the A-FAL population as an outlier, and the other westernmost populations, located near the Lake Randsfjorden, being more closely related to each other and relatively closely related to the southernmost (B-SØR) population. The eastern populations were differentiated from the western populations, whereas they were closely related to each other, and were admixed between the eastern river systems. The C-LEI and C-KAM populations were especially closely related, and the genetic differentiation, expressed both as the *F*_ST_ and *Nei*D indices, was non-significant, which was different from all the other pairs tested. This leads to a suspicion that at least one of these populations was stocked by humans in relatively recent time, as pronounced structuring is commonly found among populations of landlocked freshwater pearl mussels [27].

Natural occurrence of freshwater pearl mussels presupposes a past immigration of salmon or brown trout as hosts, and in the western populations (A and B), the freshwater pearl mussel was probably hosted by anadromous salmonids in locations connected to the sea. The invasion of freshwater fish to the Glomma river system may have followed two different routes. The upper part of the Glomma River once drained to Lake Vänern in Sweden, via the Vrangselva River, and was colonized by freshwater organisms originating from the ancient freshwater Lake Ancylus [41], which once covered the Baltic Sea and parts of Sweden [66]. Brown trout, potentially hosting mussel larvae, invaded the Glomma River through this route, until the river broke through land barriers to the west and closed the connection to Lake Vänern. Since then, the Glomma River has run to the eastern side of the Oslo Fjord, and immigrants originated from the freshwater Lake Ancylus/Vänern were spread to lower parts of the Glomma River, and could also enter the Lake Mjøsa and its tributaries. The distribution of several fish species supports this invasion theory, with one example being the Arctic grayling (*Thymallus thymallus* L.), which has a low tolerance to salinity [67] that prevents it from entering river systems by sea migration, being present only in the Glomma river system and in systems farther east. Other species with similar distribution patterns are alpine bullhead (*Cottus poecilopus*) and burbot (*Lota lota*).

The differentiation between the western and the eastern populations suggests that the populations originated from different sources, at least in part. The mussels in the western river systems were probably introduced by anadromous brown trout or salmon at some stage after the glaciation, when the locations were available for ascending anadromous fish [41]. In the eastern river systems, the species may have been introduced by landlocked brown trout and salmon (still present in Lake Vänern), originating from the ancient freshwater Lake Ancylus; however, at some stage of the land rising, all locations below 200 m a.s.l. were physically available for anadromous fish. This leaves a possibility for the anadromous origin of salmonids hosting mussels in all river systems. Nevertheless, it remains an unanswered question as to when the invasion of mussels took place. The presence of cluster 2 in the populations A-LOM, A-ETN, and B-SØR suggested a partly common origin of three of the western populations and the eastern populations, whereas the A-FAL population, as well as cluster 1, likely originated from an earlier invasion.

### 4.2. Genetic Diversity

The genetic diversity of the study populations was substantially higher in the western (*A*_R_ = 2.59–3.23 and *H*_E_ = 0.321–0.524) than in the eastern (*A*_R_ = 1.21–1.64 and *H*_E_ = 0.037–0.139) populations, and only one population, B-SØR, had genotypes in HWE and non-significant *F*_IS_. On the basis of comparative studies in central and southern Europe, researchers generally characterized *A*_R_ < 2 and *H*_E_ < 0.1 as low [27,28], and except for two populations with heterozygosity > 0.1, all the eastern populations fall into the category of low diversity. Karlsson and Larsen [68] found *A*_R_ = 2.5 and *H*_E_ = 0.377 in Fallåa (A-FAL) (on the basis of different SSR markers), and a substantially lower *A*_R_ = 1.2 and *H*_E_ = 0.065 in Hunnselva (C-HUN), confirming a low genetic diversity of mussels in the Glomma drainage.

Mussel populations depending on landlocked hosts have low genetic diversity and are found to be strongly structured [27], as was the case in this study. Bottleneck events, suggested for all the populations, result in loss of low frequency alleles, and subsequent genetic drift leads to differentiation between populations, and in addition, low population density leads to more frequent self-fertilizing and thereby reduced heterozygosity [27]. This may explain the low diversity of the landlocked eastern populations.

In a study on freshwater pearl mussels in river systems with anadromous brown trout and salmon as host species in northern Sweden, *A*_R_ was > 3 and *H*_e_ > 0.5 in 8 of 14 samples, and the genetic diversity increased downstream in the rivers [69]. If the mussel populations (C and D) of the Glomma and the Vrangselva river systems have a common immigration history with origin in tributaries to Lake Ancylus or Lake Vänern, D-FIN would be geographically closest to the source population, potentially causing the higher number of polymorphic loci in this population. Further, there were three private alleles and *A*_R_ = 1.64, compared to two or less private alleles and *A*_R_ ranging from 1.21 to 1.44 in the other eastern populations.

The isolated westernmost A-FAL population was special, being characterized with low abundance and poor recruitment [14] but with exceptionally high genetic diversity, suggesting a former large and well-reproducing parental population. The population also had low *F*_IS_ and a low proportion of siblings as compared with the eastern populations.

The negative correlation of maximum shell length and *F*_IS_ suggests an effect of inbreeding depression in the eastern populations, corresponding to what Zheng et al. [31] experienced with American bay scallops in breeding experiments. Nevertheless, the inbred C-LEI and C-KAM populations (*F*_IS_ ≥ 0.448, *H*_O_ ≤ 0.067) were characterized as abundant and well-reproducing, with the second and third highest status score (15 and 18) of all the eastern populations [14,45]. The C-KAM population included thousands of individuals [47], and recruitment had taken place during the last 20 years, coinciding with lime treatment of an acidified upstream lake. Likewise, the D-LØV population, with low genetic diversity, had successful reproduction in the 1990s when upstream lakes were lime treated [47], suggesting that reproduction was hampered by acidic water.

Suboptimal water quality in several mussel locations, and apparently positive effects of limestone powder on mussel recruitment in some of them, suggested that liming may be necessary to secure the freshwater pearl mussels’ existence in some of the study locations [45]. When pH is about 6.0 and episodically lower and with [Ca] < 2.0 mg/L, it probably affects freshwater pearl mussel recruitment negatively. This interpretation is supported by the results of a liming project in a stream of similar quality, bringing pH above 6.2 and [Ca] above 2.5 mg/L, resulting in recruitment recovery of a freshwater pearl mussel population in Southwest Norway [70].

No negative effects of inbreeding on recruitment or viability were indicated when comparing genetics and recruitment of the study populations. It may be hypothesized that the general low genetic diversity of the eastern populations is due to an origin from settlers hosted by landlocked salmonids, different from the western populations descending from mussels hosted by anadromous host fish. There was no indication of selection on the analyzed markers, but SSRs are supposed to be neutral and not subject to selection, although there are exceptions [71]. Inbreeding in populations exposed to selection pressure may result in purging of harmful genes, favoring those conferring beneficial traits [72]. The present genotypes of the eastern populations may therefore in part be a result of selection caused by environmental stress, resulting in purging of genes in former generations, and efforts to preserve the remaining genetic diversity are urgent. Each population, with a possible exception of the C-LEI and C-KAM, should be regarded as unique ESUs, until eventually inbreeding should appear to affect the population viability. Then the eastern populations could be pooled to increase genetic diversity by experimentally admixing the populations in hatcheries. The viability of the admixed offspring should be tested by stocking in the eastern river systems in locations with suitable physical and chemical environments, but at present are not inhabited by the mussel. If this succeeds, an admixed population may be kept in captivity as a reserve stock.

## 5. Conclusions and Recommendations

Recruitment failure, low genetic diversity, and a high portion of siblings in the majority of the study populations suggested that they are prone to further loss of genetic variation and may be threatened by extinction in the future, i.e., within 100 years, except for the B-SØR, which appeared to be a healthy population. Habitat conservation is necessary, and in several study locations, the water quality, expressed as pH and [Ca^2+^], is suboptimal for pH-sensitive organisms such as freshwater pearl mussels. Water quality and mussel reproduction in the study streams should be monitored, and lime treatment should be considered.

Excess of homozygotes and significant inbreeding coefficients suggested inbreeding, with a possible risk of inbreeding depression and future negative effects on reproductive performance [32]. Restoration measures must be based on the local populations of freshwater pearl mussels and host brown trout as the infection rate of mussel larvae depends on genetic predisposal [73,74]. Some of the eastern populations, all representing cluster 2, could be pooled if the low genetic variation and inbreeding appears to hamper recruitment and viability.

## Figures and Tables

**Table 1:** Physical and chemical variables at the sampling sites, minimum–maximum scale length (*L*) in our samples, and status score ^1^ of the mussels of the sampling streams. Labels A–D in the abbreviation indicate the main river system of the locations.

Stream	Abbreviation	Alt.	pH	Conductivity	Color	Ca	*L* mm	Status
		m a.s.l.		mS m^−1^	mg Pt L^−1^	mg L^−1^	Min–Max	Score ^1^
Lomsdalselva	A-LOM	170	5.5 ^2^–6.4	1.6	70	1.7	79–131	8
Etna	A-ETN	143	6.8	3.1	20	5.6	44–125	6
Fallselva	A-FAL	304	6.5	2.4	52	3.5	55–115	9
Sørkedalselva	B-SØR	150	7.0	2.8	43	5.6	84–116	22
Hunnselva	C-HUN	300	7.6	11.5	36	16	80–115	7
Leira	C-LEI	240	5.3 ^2^–6.4	1.7	22	1.9	63–101	18
Kampåa	C-KAM	185	5.3 ^2^–6.8	2.7	68	3.2	62–107	15
Gjerda	C-GJE	267	6.0 ^3^–6.1	2.8	68	1.8	78–101	6
Bråtåa	D-BRÅ	160	5.7 ^3^–6.4	3.5	72	3.3	65–114	8
Finnsrudåa	D-FIN	150	6.1	4.1	40	4.2	61–118	20
Løvhaugsåa	D-LØV	293	5.6 ^2^–6.0	2.1	78	1.4	86–116	6

^1^ = from [14], ^2^ = measured before liming, ^3^ = measured in spring.

**Table 2:** Pairwise genetic differentiation of the 11 study populations expressed as *F*_ST_ (below diagonal) and unbiased Nei’s differentiation index (*Nei*D, above diagonal)

	A-LOM	A-ETN	A-FAL	B-SOR	C-HUN	C-LEI	C-KAM	C-GJE	D-BRA	D-FIN	D-LOV
A-LOM	-	0.149	0.638	0.181	0.198	0.136	0.135	0.206	0.125	0.195	0.189
A-ETN	0.099	-	0.624	0.170	0.222	0.139	0.144	0.191	0.161	0.215	0.225
A-FAL	0.199	0.209	-	0.770	0.819	0.891	0.889	0.673	0.816	0.834	0.788
B-SOR	0.120	0.113	0.266	-	0.178	0.109	0.094	0.189	0.142	0.168	0.153
C-HUN	0.172	0.188	0.370	0.155	-	0.077	0.080	0.104	0.098	0.038	0.163
C-LEI	0.154	0.157	0.391	0.137	0.134	-	**0.001**	0.179	0.090	0.065	0.068
C-KAM	0.155	0.159	0.390	0.115	0.149	**0.013**	-	0.168	0.084	0.068	0.063
C-GJE	0.157	0.153	0.307	0.173	0.170	0.317	0.247	-	0.099	0.164	0.208
D-BRA	0.121	0.142	0.332	0.148	0.198	0.256	0.197	0.160	-	0.078	0.123
D-FIN	0.162	0.175	0.346	0.136	0.053	0.094	0.101	0.172	0.127	-	0.122
D-LOV	0.170	0.183	0.331	0.148	0.267	0.150	0.139	0.285	0.239	0.166	-

**Bold face** = non-significant.

**Table 3:** Genetic diversity of the 11 study populations based on 11 markers, number of individuals analysed (*n*), number of alleles per locus (*A*_L_), number of private alleles (*A*_P_), number of polymorphic loci (*P*_L_), inbreeding coefficient (*F*_IS_) (* = significant), and expected unbiased heterozygosity (*H*_E_).

Population	*n*	*A*_L_	*A*_P_	*P*_L_	*F*_IS_	*H*_E_
A-LOM	30	3.6	11	11	0.122 *	**0.405**
A-ETN	8	2.8	1	11	0.261 *	**0.404**
A-FAL	30	4.1	9	11	0.143 *	**0.586**
B-SØR	24	3.	5	10	0.110	0.392
C-HUN	41	1.4	1	4	0.579 *	**0.176**
C-LEI	32	1.4	0	4	0.847 *	**0.099**
C-KAM	28	1.5	3	4	0.448 *	**0.120**
C-GJE	29	1.8	2	4	0.801 *	**0.385**
D-BRÅ	22	1.5	0	4	0.588 *	**0.422**
D-FIN	30	2.1	3	7	0.575 *	**0.195**
D-LØV	29	1.6	2	4	0.699 *	**0.420**

**Boldface** = significantly different from observed heterozygosity.

**Table 4:** Estimated number of pairs of full siblings and proportion of full siblings in percentence of possible pairs, and maximum family group detected; *M* = mean Garza–Williamsons modified index.

Population	FullSib	% FullSib	Max. Family	*M*	
			Group Size	Mean	Min–Max
A-LOM	23	5.3	6	0.23	0.04–0.80
A-ETN	0	0	0	0.16	0.04–0.50
A-FAL	2	3.5	3	0.20	0.03–0.70
B-SØR	12	4.4	5	0.18	0.02–0.60
C-HUN	233	28.4	22	0.07	0.02–0.25
C-LEI	416	83.8	29	0.07	0.02–0.25
C-KAM	220	58.2	28	0.09	0.02–0.40
C-GJE	23	15.3	11	0.11	0.02–0.40
D-LØV	17	4.2	4	0.08	0.02–0.30
D-BRÅ	35	15.2	6	0.10	0.02–0.25
D-FIN	221	50.8	27	0.08	0.02–0.25

## Supplementary Materials

The following are available online at https://www.mdpi.com/2073-4425/11/9/1061/s1, Table S1: Criteria and scores (1-6 points) for assessing status/viability of freshwater pearl mussel. Reworked by Larsen & Magerøy [14] after Söderberg [75].; Table S2. Characteristics of six simple sequence repeat (SSR) loci for the freshwater pearl mussel (*Margaritifera margaritifera*): Locus designation, repeat motif, primer sequences, optimal annealing temperature (*T*_a_), number of observed alleles (*N*_A_) and allele size range. Table S3. GenePop.format.txt.
