# Supplementary material for: Assessing Evolutionary Significant Units (ESU) of the Endangered Freshwater Pearl Mussel (Margaritifera margaritifera) in Southeast Norway on the Basis of Genetic Analysis
Source: Genes (Basel). 2020 Sep 8;11(9):1061. doi: 10.3390/genes11091061 (PMC7565849; doi:10.3390/genes11091061)
Supplement: Supplementary file 1 [file genes-11-01061-s001.zip › Table S1.docx]

**Table S1.** Criteria and scores (1-6 points) for assessing status/viability of freshwater pearl mussel. Reworked by Larsen & Magerøy [1] after Söderberg [2].

|  | | Points | | | | | |
| --- | --- | --- | --- | --- | --- | --- | --- |
| Criteria | | 1 | 2 | 3 | 4 | 5 | 6 |
| 1 | Population size (●1000) | < 5 | 5-10 | 11-50 | 51-100 | 101-200 | >200 |
| 2 | Mean density (N/m^2^) | < 2 | 2.1-4 | 4.1-6 | 6.1-8 | 8.1-10 | >10 |
| 3 | Populated stretch (km) | < 2 | 2.1-4 | 4.1-6 | 6.1-8 | 8.1-10 | >10 |
| 4 | Smallest specimen found (mm) | >50 | 41-50 | 31-40 | 21-30 | 11-20 | ≤10 |
| 5 | Proportion of specimens < 2 cm (%) | >0-1 | >1-2 | >2-3 | >3-4 | >4-5 | >5 |
| 6 | Proportion of specimens < 5 cm (%) | >0-5 | 6-10 | 11-15 | 16-20 | 21-25 | >25 |

**References**

1. Larsen, B. M., and Magerøy, J. H. Elvemuslinglokaliteter i Norge - En beskrivelse av status som grunnlag for arbeid med kartlegging og tiltak i handlingsplanen for 2019–2028. Trondheim: Norwegian Institute of Nature Research, 2019.

2. Söderberg, H. Undersökningtyp: Övervakning av flodpärlmussla. In *Flodpärlmusslan i Sverige.*, edited by M.O.G. Eriksson, Henrikson, L. & Söderberg, H. Stockholm: Naturvårdsverket, 1998.
