# Supplementary material for: Assessing Evolutionary Significant Units (ESU) of the Endangered Freshwater Pearl Mussel (Margaritifera margaritifera) in Southeast Norway on the Basis of Genetic Analysis
Source: Genes (Basel). 2020 Sep 8;11(9):1061. doi: 10.3390/genes11091061 (PMC7565849; doi:10.3390/genes11091061)
Supplement: Supplementary file 1 [file genes-11-01061-s001.zip › Table S2. Characteristics of six simple sequence repeat (SSR) loci for the freshwater pearl mussel (Margaritifera margaritifera).docx]

**Table S2.** Characteristics of six simple sequence repeat (SSR) loci for the freshwater pearl mussel (*Margaritifera margaritifera*): Locus designation, repeat motif, primer sequences, optimal annealing temperature (*T*_a_), number of observed alleles (*N*_A_) and allele size range.

| Locus | Repeat motif | Primersquences (5` →3`) | *T*_a_, °C | *N*_A_ | | Allele size range (bp) | |
| --- | --- | --- | --- | --- | --- | --- | --- |
| *Mm2209* | (ATTT)_13_ | F:GGCACCTTCTTGGTAATTCT  R:CCTTCACCTGATACTCACAC | 55 | 7 | 183-219 | |  |
| *Mm2230* | (CTAC)_10_ | F:ATGACGTCCCGATAAACTG  R:CATTGTACCCTCTCTCACGA | 55 | 3 | 238-246 | |  |
| *Mm2233* | (ATT)_14_ | F:GATGAGGAACGGTATAGGG  R:TACAGCAGCAAGAGACTAAC | 55 | 2 | 162-165 | |  |
| *Mm2235* | (TATG)**_15_** | F:AGTTTGAGGATGGGTTTTG  R:TTTACCTTTAGTTTGGCACC | 55 | 10 | 135-239 | |  |
| *Mm2236* | (ATCT)_14_ | F:CCAGAACTGCGATTAAATGA  R:GGGAAAAGCTGCAAACTAA | 55 | 5 | 183-231 | |  |
| *Mm2238* | (TGG)_10_ | F:GAAAAATCGGTATGGGGTTAG  R:TTTACGACGAAGCTGCAC | 55 | 3 | 118-212 | |  |
